# Salinomycin as a potent anticancer stem cell agent: State of the art and future directions

**DOI:** 10.1002/med.21870

**Published:** 2021-11-16

**Authors:** Dan Qi, Yunyi Liu, Juan Li, Jason H. Huang, Xiaoxiao Hu, Erxi Wu

**Affiliations:** ^1^ Department of Neurosurgery Baylor Scott & White Health Temple Texas USA; ^2^ Neuroscience Institute Baylor Scott & White Health Temple Texas USA; ^3^ Molecular Science and Biomedicine Laboratory, State Key Laboratory for Chemo/Biosensing and Chemometrics, College of Biology, College of Chemistry and Chemical Engineering, Collaborative Innovation Center for Molecular Engineering for Theranostics Hunan University Changsha China; ^4^ Department of Surgery Texas A&M University College of Medicine Temple Texas USA; ^5^ Shenzhen Research Institute Hunan University Shenzhen Guangdong China; ^6^ LIVESTRONG Cancer Institutes and Department of Oncology, Dell Medical School The University of Texas at Austin Austin Texas USA; ^7^ Department of Pharmaceutical Sciences Texas A&M University College of Pharmacy College Station Texas USA

**Keywords:** anticancer stem cell agent, salinomycin, drug binding target, drug derivatives, nano‐drug delivery

## Abstract

Cancer stem cells (CSCs) are a small subpopulation of cells within a tumor that can both self‐renew and differentiate into other cell types forming the heterogeneous tumor bulk. Since CSCs are involved in all aspects of cancer development, including tumor initiation, cell proliferation, metastatic dissemination, therapy resistance, and recurrence, they have emerged as attractive targets for cancer treatment and management. Salinomycin, a widely used antibiotic in poultry farming, was identified by the Weinberg group as a potent anti‐CSC agent in 2009. As a polyether ionophore, salinomycin exerts broad‐spectrum activities, including the important anti‐CSC function. Studies on the mechanism of action of salinomycin against cancer have been continuously and rapidly published since then. Thus, it is imperative for us to update its literature of recent research findings in this area. We here summarize the notable work reported on salinomycin's anticancer activities, intracellular binding target(s), effects on tumor microenvironment, safety, derivatives, and tumor‐specific drug delivery; after that we also discuss the translational potential of salinomycin toward clinical application based on current multifaceted understandings.

**Abbreviations**
AM5: ionomycin
AZT: 3'‐azido‐3'‐deoxythymidine
Bz: benzoyl group
CD: cluster of differentiation
c‐Myc: avian myelocytomatosis virus oncogene cellular homolog
CSC: cancer stem cell
Cyto c: cytochrome c
DARTS: drug affinity responsive target stability
DDS: drug delivery system
EGCG: epigallocatechin gallate
EGFR: epidermal growth factor receptor
EMT: epithelial–mesenchymal transition
ER: endoplasmic reticulum
EC_50_
: the half maximal effective concentration
FDA: the United States Food and Drug Administration
FdU or 5‐FU: floxuridine
hEGR: human ether‐a‐go‐go‐related gene
HSPC: hematopoietic stem/progenitor cells
IC_50_
: the half maximal inhibitory concentration
IP: immunoprecipitation
IRP2: iron‐responsive element‐binding protein 2
JAK: Janus kinase
Klf4: kruppel‐like factor 4
LD_50_
: lethal dose, 50%
LEF: lymphoid enhancer factor
MDR: multidrug resistance
MoA: mechanism/mode of action
MON: monensin
MSC: mesenchymal stem cell
NB: neuroblastoma
NBD: nitrobenzoxadiazole
NCL: nucleolin
NMR: Nuclear Magnetic Resonance
NP: nanoparticle
OCT4: octamer‐binding transcription factor 4
PBMC: peripheral blood mononuclear cell
PEG: polyethylene glycol
PLGA: poly(lactic‐co‐glycolic acid)
ROS: reactive oxidative species
SAL: salinomycin
SAR: salinomycin derivative
SARS‐CoV‐2: severe acute respiratory syndrome coronavirus 2
SAX: salinomycin conjugate
SI: selectivity index
SOX2: sex determining region Y‐box 2
STAT: signal transducer and activator of transcription
TCF: T cell factor
TF: transcription factor
TAM: tumor associated macrophage
TIC: tumor initiating cell
TME: tumor microenvironment.

## 1 INTRODUCTION—CANCER STEM CELLS (CSCs) AND SALINOMYCIN

In recent years, cancer research has undergone a paradigm shift, moving from broad proliferative suppression toward mechanistic research on causes that trigger cancer or drive malignancy as well as therapy resistance. Accumulating studies support the exciting notion that there is a small population of cancer cells hidden in the tumor bulk which are able to initiate tumor growth and remodel tumor cell population against treatment. At the outset, these cells were called tumor initiating cells (TICs) and later named stem‐like cancer cells or CSCs. CSCs are renowned for their indefinite potential of self‐renewal and generation of heterogeneous tumor cells.^1^, ^2^, ^3^, ^4^ Studies have suggested that CSCs can be plastic and transform between quiescence state and active proliferation state.^5^, ^6^ The induction of stemness‐related genes and oncogenes, such as sex determining region Y‐box 2 (SOX2), octamer‐binding transcription factor 4 (OCT4), kruppel‐like factor 4 (Klf4), and avian myelocytomatosis virus oncogene cellular homolog (c‐Myc), in nontumorigenic mammary epithelial cells, has been shown to be capable of transforming the original cells into tumorigenic CSCs, lending a strong support to the plasticity of CSCs.^7^ The tumor microenvironment (TME) shows a dominant regulatory role in tumor formation, progression, and distant migration.^8^, ^9^, ^10^ CSCs may also be regulated by stromal cells and immune cells in the TME or regulate the architecture of TME and thereby shape the heterogeneous tumor bulk, which contributes back to the CSC plasticity.^5^, ^11^, ^12^, ^13^, ^14^

The existence of CSCs is considered to be one of the major reasons for tumor recurrence and therapy resistance. Current hypotheses regarding CSCs' origin include: (1) CSCs may be generated from normal stem cells or progenitor cells^15^ after mutations occur^3^, ^16^ or after escaping regulation^4^, ^15^; (2) CSCs may originate from mesenchymal stem cells during wound healing to repair damaged tissues^11^; (3) CSCs may arise from normal somatic cells after rendered stem‐like characteristics, for instance, through epithelial–mesenchymal transition (EMT) or aberrant epigenetic regulation.^16^, ^17^, ^18^ However, the explicit mechanism of CSC generation could be distinct in various cancer types or in different patients and is yet to be fully understood. Current approaches to identify CSCs within a tumor bulk are mainly classified into two: (1) by an antigenic method from cell surface marker expression,^19^ such as cluster of differentiation (CD)133, CD44, and nestin, that are also recognized as markers expressed in normal stem cells; (2) by a functional method from their ability to efflux DNA‐binding dyes using flow cytometry, and may show a higher expression of membrane pumps such as ABCG2 (adenosine triphosphate‐binding cassette subfamily G member 2), ABCA3 (adenosine triphosphate‐binding cassette transporter A3) involved in multidrug resistance (MDR). However, due to the heterogeneity of the CSC population and the complexity of CSC regulation (i.e., plasticity),^20^ a standard in vitro protocol or definition to precisely identify CSCs has not been well established other than their tumorigenic ability in xenograft animal models. Nonetheless, mounting efforts have been made by global researchers trying to deepen the understanding of CSCs and explore the ways to target them. We have recently summarized research advances for breast CSCs, one of the most studied cancer types, and enumerated CSC‐associated multifold intercellular and intracellular interplays.^21^

**Figure 1:**
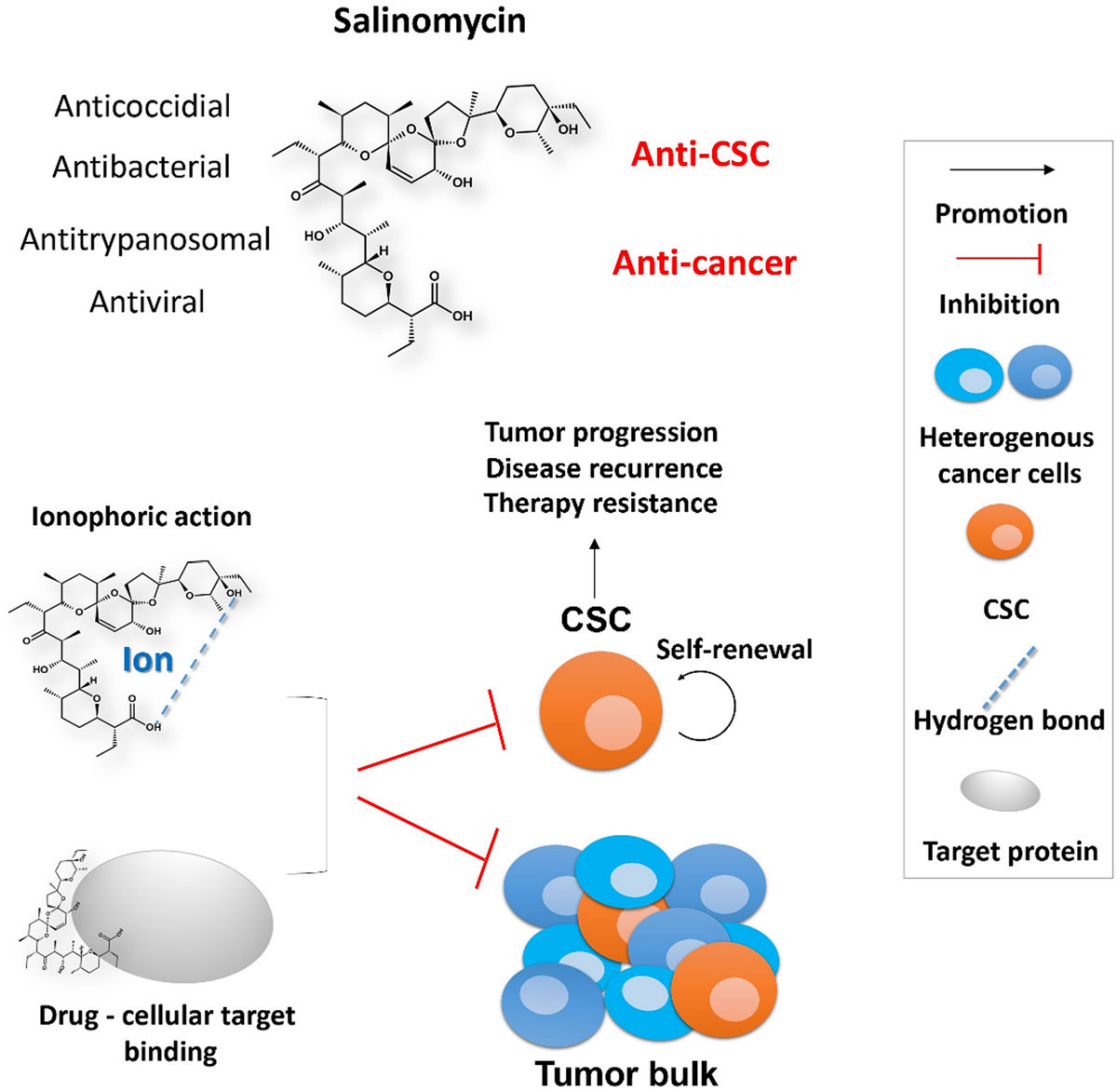
Cancer stem cell and salinomycin's activities [Color figure can be viewed at wileyonlinelibrary.com]

Shibue and Weinberg have pointed out that most conventional therapeutics lack an efficiency in eradicating carcinoma cells that have entered the CSC state, which thereby permits a CSC‐dependent disease relapse.^18^ Therefore, novel drugs and regimens targeting CSCs would have a potential clinical relevance for cancer treatment, such as by inducing cell death of CSCs, by inhibiting CSC‐associated cell signaling pathways (e.g., Wnt, Notch, Hedgehog, and Hippo pathways), by reversing EMT or by immuno‐approaches targeting CSC cell surface markers.^18^, ^22^, ^23^ Indeed, it is Weinberg group who first reported that salinomycin (Figure 1), a monocarboxylic polyether chemical, has anti‐CSC activity in breast cancer in 2009.^24^ Research efforts focusing on salinomycin's function and mechanism of eliminating CSCs have been continuously reported since then. Aligning with Gupta's work, Ginestier group recently performed a genome‐wide RNA interference (RNAi) screening aiming to uncover important genes regulating the breast CSC fate. They then tested a panel of compounds for their inhibitory effects on the identified CSC‐fate regulators, and salinomycin stood out again as one of the three most potent effectors.^25^

Salinomycin is originally applied as an antibacterial and anticoccidial drug, which is commercially used as a coccidiostat for poultry.^26^, ^27^ The potential antidote activity of salinomycin has also been documented from murine experiment results.^28^, ^29^ Several studies have further revealed that salinomycin could be an attractive drug candidate against viruses, for example, it is repurposed recently as a potential anti‐severe acute respiratory syndrome coronavirus 2 (SARS‐CoV‐2) agent.^30^ The mechanism of action (MoA) of salinomycin against microbes is likely because of its ionophoric nature.^31^ In cancer therapy research, salinomycin is a selective anti‐CSC agent and a potent partner in cotherapies of cancer, as it has been shown to sensitize radiation and many clinically used chemodrugs such as doxorubicin, trastuzumab, gemcitabine, temozolomide, and tamoxifen.^32^, ^33^, ^34^, ^35^, ^36^, ^37^, ^38^ We have previously summarized the biological activities of salinomycin published before 2013 and predicted potential challenges on using salinomycin as an anticancer agent.^27^ However, the cellular functional binding target of salinomycin was unknown by then. Additionally, the attempts on maximizing drug efficacy via nanocarrier‐based delivery system have been published since then. The chemical modifications to help either uncover salinomycin's function or enhance its efficacy are also in the bench state. Besides, more profound studies on dissecting the CSC‐killing activities of salinomycin and the preclinical application have been reported. Therefore, it is time herein for us to update again the recent important advances of the preclinical studies on the anti‐CSC mechanisms, safety and toxicity, chemical derivatives, nano‐carrier‐based drug delivery systems, and clinical application cases of salinomycin.

## 2 MULTIFUNCTIONAL MECHANISMS OF SALINOMYCIN AGAINST CSC

**Figure 2:**
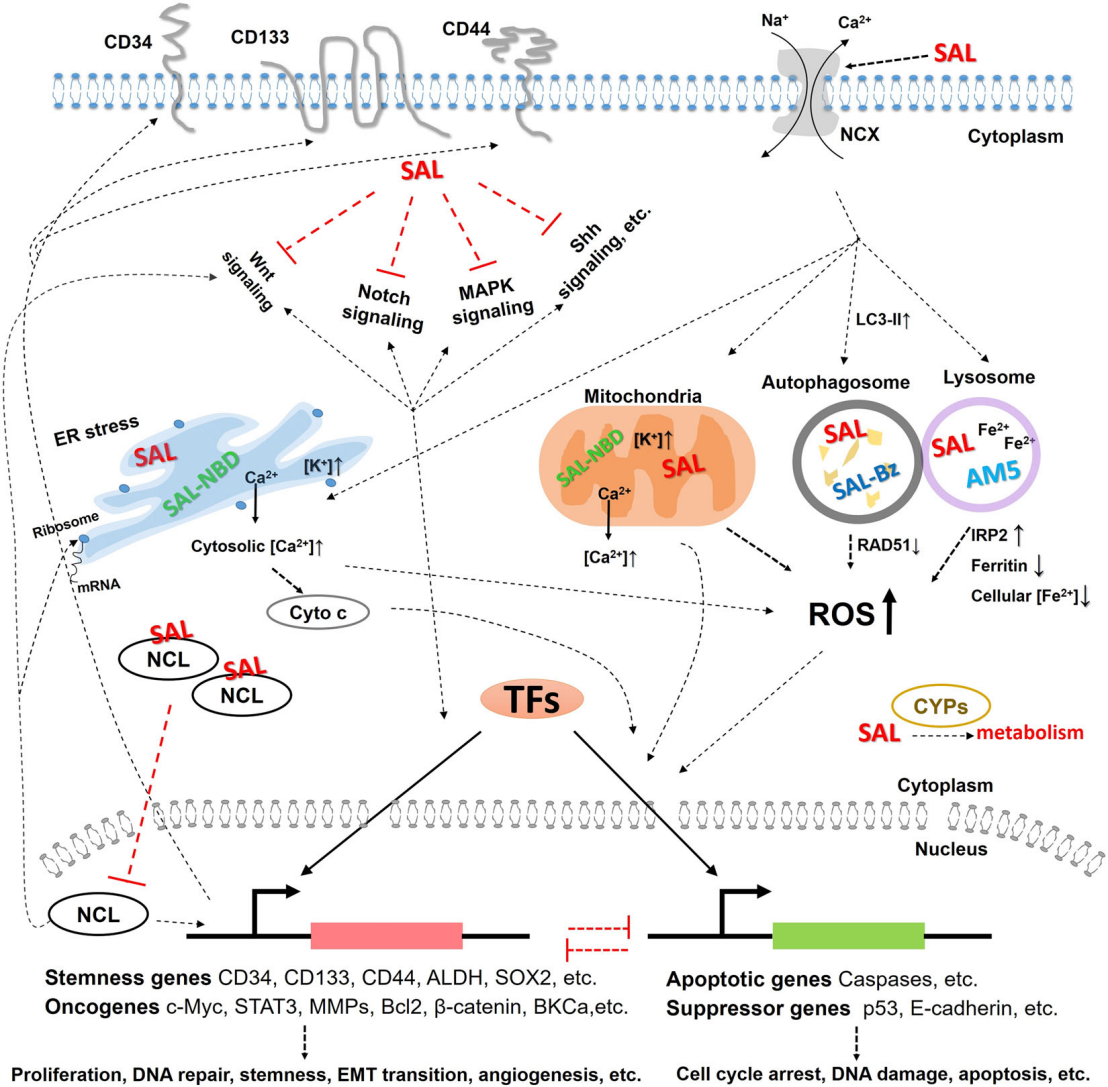
Possible mechanisms of salinomycin's anticancer effects. Cyto c, cytochrome c; EMT, epithelial–mesenchymal transition; IRP2, iron‐responsive element‐binding protein 2; MMPs, matrix metallopeptidases; TFs, transcription factors [Color figure can be viewed at wileyonlinelibrary.com]

Although the explicit molecular mechanism of salinomycin's suppression activity on CSCs remains unknown, evidence has accumulated, telling that the biological actions of salinomycin on eliminating CSCs may be manifold and cell context‐dependent. So far, the reported MoAs of salinomycin eliminating CSCs include: the modulation of multiple signaling pathways in cancer cells, such as inhibition of Wnt and mitogen‐activated protein kinase (MAPK) pathways,^27^, ^39^, ^40^ the initiation of autophagy,^41^, ^42^ the decrease of adenosine triphosphate (ATP) levels along with the elevation of reactive oxygen species (ROS) production,^43^ the sequestration of iron in lysosomes,^44^ the triggering of DNA damage and prevention of DNA repair,^32^, ^38^, ^45^, ^46^ the induction of endoplasmic reticulum (ER) stress,^47^, ^48^, ^49^, ^50^ and more recently from our own research—the suppression of CSC marker expression via the interaction with its cellular binding target nucleolin (NCL).^51^ We summarize below the current eye‐catching work focused on the molecular actions of salinomycin in terms of its anti‐CSC activity (Figure 2).

### 2.1 Functionality as a polyether ionophore and effects on mitochondria

Salinomycin (molecular formula C_42_H_70_O_11_) was characterized as a new member of polyether ionophore antibiotics in 1973–1974 by Kinashi et al.^52^ and Miyazaki et al.^31^ Due to the difficulty of directly identifying solid‐state salinomycin by X‐ray, Kinashi and Otake analyzed and published its p‐iodophenacyl ester derivative's structure.^52^ Miyasaki and colleagues then found that salinomycin is a monovalent and divalent cation (Na^+^, K^+^, Ca^2+^, Rb^+^, etc.) transporter and exerts K^+^ preference toward organic phase in a two‐phase system. Salinomycin's wide‐ranged antimicrobial activities were determined subsequently including effects against protozoa, gram‐positive bacteria such as mycobacteria, and some filamentous fungi.^27^, ^53^ Riddell et al. reported that salinomycin mediates the transport of Na^+^ and K^+^ ions through phospholipid bilayers using ^23^ Na‐and ^39^ K‐NMR (nuclear magnetic resonance) spectroscopy.^54^, ^55^ Subsequent studies by Vértesy group in the 1990s published salinomycin sodium's X‐ray structures in both solution‐state and solid‐state, claiming its functional role as a cation ionophore and structure–activity understandings.^56^, ^57^ The mechanism of salinomycin against parasites and trypanosoma has been demonstrated as its ability to increase Na^+^ influx and induce cell swelling, which has been later suggested may not be the same mechanism for its anticancer action.^58^ In Mai et al.'s work, they observed increased Na^+^ influx at 20‐fold high concentration of the dose inhibiting breast CSCs.^44^ A few years ago, Huczyński briefly summarized the possible mechanism of polyether ionophore and proposed three models of monovalent and divalent transportation across lipid membrane.^59^

The potassium ion channel has been known to alter mitochondrial function.^60^, ^61^ In 1976, Miyazaki and colleagues first investigated the effect of salinomycin on mitochondrial ion translocation and respiration and reported that 0.4 μM salinomycin could release K^+^ from mitochondria isolated from rat liver under the condition of K^+^ preloaded by K^+^ uptake stimulators.^55^ Meanwhile, it could inhibit the oxidation of glutamate, α‐ketoglutarate, malate, and pyruvate but not that of β‐hydroxybutyrate or succinate. Salinomycin reversed the cell swelling induced by K^+^ uptake stimulators and suppressed the oxidative phosphorylation in mitochondria without substrate specificity. The pretreatment of salinomycin could prevent K^+^ uptake in mitochondria. Notably, salinomycin blocked the mitochondrial retention of K^+^ more effectively than that of Na^+^. It inhibited respiration in medium with low but not high K^+^ concentration even with the addition of K^+^ uptake stimulators.^55^ This study suggests that the transport of cation by salinomycin can be condition‐dependent, and could be affected by ion gradients.

A later study by Managò et al. investigated the early effect of salinomycin as a K^+^ ionophore on mitochondrial function in human cells in vitro.^62^ They introduced two other K^+^ ionophores, including one of the K^+^ uptake stimulator valinomycin to seek for the possible MoA of salinomycin. As a result, salinomycin decreased respiration at tens of minutes without inducing ROS production and showed a similar activity to that of the K^+^/H^+^ exchanger nigericin. As expected, a strong cytotoxicity and an apoptotic effect of salinomycin on leukemia cells was observed. Additionally, B‐cell lymphoma 2 (Bcl‐2)‐associated X protein (Bax)‐dependent apoptosis was induced in Bax/Bak (Bcl‐2 homologous antagonist/killer)‐less mouse embryonic fibroblasts in a dose‐dependent manner. Mesenchymal stromal cells somehow attenuated the apoptotic effect of salinomycin in lymphoma cells, suggesting TME may affect salinomycin's potency.^63^ Altered mitochondrial membrane potential was observed early after salinomycin (1 μM) treatment.^63^ Meanwhile, as for human primary fibroblasts, mesenchymal stromal cells and healthy B cells, salinomycin showed slight toxicity after the dose was increased. This study supports the direct influence of salinomycin on mitochondria as a K^+^/H^+^ antiporter, and partially explains the various effects of salinomycin on different cancer cell types maybe because of distinct metabolic states. In addition, Qin et al. reported that salinomycin treatment led to p53 translocation to mitochondria and causes necrosis of glioma cells in vitro.^64^

### 2.2 Induction of autophagy, ROS, and DNA damage

Autophagy plays a duel role of both protecting and inhibiting cell survival in cancer cells: on one hand, it can inhibit ROS generation and protect cells from undergoing apoptosis; on the other hand, Li et al. showed that salinomycin treatment increased the expression of microtubule‐associated protein 1A/1B‐light chain 3 (LC3), suggesting the induction of autophagy.^65^ Salinomycin treatment also elevated the intracellular ROS level, which resulted in cell death at least partially through the caspase‐dependent apoptosis.^66^ Knockdown of autophagy protein 7 (ATG7) partially blocked the salinomycin‐induced cell death, indicating that it triggered the autophagy‐related apoptosis.^67^ Yue et al. later demonstrated that salinomycin blocked the autophagy flux and the lysosomal proteolytic activity in both CSCs and non‐CSCs derived from breast cancer cells.^68^ They previously showed that autophagy is essential in CSCs. Therefore, salinomycin eradicating CSCs could also happen through the induction of autophagy‐related apoptosis. Several other groups also reported the complex modulation of autophagy by salinomycin in cancerous cells, but a harmonized observation of intracellular oxygen species production, LC3/autophagy induction, and subsequent cell death under single‐drug treatment in vitro.^38^, ^41^, ^42^, ^69^

A few research groups, including us, observed that salinomycin itself or by sensitizing chemodrugs or radiation, moderately or strongly increases DNA damage, inducing G2 arrest and reduction of p21 protein level in cancer cells.^45^, ^46^, ^70^, ^71^, ^72^ In accord with these findings, we also further noticed the phosphorylation of tripartite motif‐containing 28 (TRIM28) along with salinomycin treatment in neuroblastoma cells (unpublished data). This phosphorylation has been well known to be associated with DNA damage response and chromatin relaxation.^73^ Zhao et al. recently found that salinomycin induced DNA damage and G1 arrest in glioblastoma (GBM) cells which were ROS—mediated,^74^ indicating that DNA damage of salinomycin treatment could be a secondary effect. More recently, by taking the advantage of RNA sequencing, Law group revealed that the drug combination of salinomycin and dasatinib (a Src kinase inhibitor) exhibited synergism through horizontal suppression of multiple pathways in breast cancer cell lines, including the estrogen‐mediated S‐phase entry pathway (G1/S arrest), and the BRCA1 (breast cancer gene 1) and DNA damage response pathway.^75^

As for glioma, Xipell et al. in 2016 reported that salinomycin could block the homologous recombination DNA repair in response to temozolomide (TMZ)‐induced DNA damage.^32^ Their results showed that salinomycin decreased the level of the DNA recombinase RAD51, the O^6^‐methylguanine‐DNA methyltransferase (MGMT) and the multidrug resistance‐associated protein (MRP). It is later confirmed also by our group that salinomycin treatment markedly inhibits the proliferation of TMZ‐resistant GBM cells,^76^ along with a dramatic expressional decrease of MRP and MGMT in GBM cell lines (unpublished data). Additionally, an Australian research group investigated the effect of salinomycin on DNA repair after radiation using in vitro cultured GBM primary tumor cells, patient ex vivo explants and orthotopic in vivo models.^38^ Their results showed that salinomycin alone induced G2 arrest in classical and mesenchymal GBM patient‐derived cells, especially in mesenchymal subtype, in accord with the original identification of salinomycin from an EMT‐induced breast cell population.^24^ Though a crude cell lysate pulldown analysis using biotin‐linked salinomycin as bait, they identified that the most bait‐associated proteins belong to nucleotides metabolism cluster. On top of that, salinomycin and its benzoylated derivative (SAL‐Bz) treatment after radiation activated LC3‐II expression and markedly decreased RAD51 expression, indicating the prevention of DNA repair, aligning with Xipell et al.'s result. Therefore, they proposed a possible autophagic route of salinomycin through which it averts DNA repair, in response to radiation‐induced DNA damage, and subsequent tumor recurrence. In orthotopic murine models, salinomycin treatment alone prolonged the overall mice survival time compared with radiation, while a combined treatment evidently extended the survival times of mice.^38^ They also generated several derivatives and tested their efficacies in parallel, which will be refined in the following summary section of salinomycin's derivative research.

### 2.3 Induction of ER stress

Li et al. showed that salinomycin induces autophagy through the activation of ER stress, the treatment of ER stress antagonist attenuated the autophagic activation in non‐small cell lung cancer cells.^65^ They found that through the mentioned route, the chemodrug sensitivity of tumor cells was potentiated. Whereas they further revealed that autophagy, which is well known as a double‐edged sword in cells, also played a protective role in salinomycin‐treated cells. The silence of autophagy‐related factors ATG5 or ATG7 increased apoptosis rates of salinomycin‐treated cells, indicating again the complexity of salinomycin's actions against cancer cells.

More recently, Strand and Colleagues have put great efforts on the uncovering of salinomycin's anti‐CSC effect including the development of experimental tools such as a fluorescent salinomycin analog conjugate, and the mechanistic investigation. They recently reported that salinomycin also induces ER Ca^2+^ release and activates ER stress in breast cancer cells.^49^, ^50^, ^77^ They showed the diffusion of a fluorescent conjugate of salinomycin (SAL‐NBD) in cytoplasm, particularly in ER and lipid droplet (LD) after treatment. An intracellular distribution pattern was also observed previously in Mai et al.'s research, while in their work a salinomycin derivative was accumulated in lysosome.^44^ It is known that the disruption of Ca^2+^ signaling activates ER stress coping responses, such as the unfolded protein response (UPR), and the mobilization of pathways to regain ER homeostasis, as the cellular Ca^2+^ homeostasis is tightly maintained to control free and bound Ca^2+^ levels in all parts of a cell.^78^ Strand group observed a concomitant activation of UPR proteins, such as C/EBP homologous protein (CHOP), G protein‐coupled receptor 78 (GPR78), and activating transcription factor 6 (ATF6), after treated with salinomycin's fluorescent conjugate. Knockdown of CHOP in a breast cancer cell line impeded the conjugate's ability to downregulate β‐catenin, a key effector in Wnt signaling.^50^ In parallel, this conjugate increases the enzymatic activity of protein kinase C (PKC) by ~30%, which is a Wnt pathway inhibitor. Therefore, as a potential calcium ionophore, salinomycin may also modulate calcium homeostasis and thereby alter Ca^2+^‐involved cellular events.

### 2.4 Suppression of Wnt signaling pathway

Lu et al. assessed the anticancer activity of salinomycin focusing on Wnt signaling pathway using both in vitro reporter system and patient samples. They found that salinomycin treatment markedly suppressed Wnt1 and its downstream β‐catenin compared with a moderate effect on Fizzled class receptor 5 (Fzd5) in vitro. Phosphorylation of lipoprotein receptor‐related protein‐6 (LRP6) which is essential in Wnt signaling is therefore decreased in response to salinomycin treatment. Given that primary chronic lymphocytic leukemia (CLL) cells produce Wnt proteins and have constitutive Wnt activation, they observed the sensitivity of CLL cells to salinomycin and 100‐fold higher cell apoptosis rate in comparison to peripheral blood mononuclear cells (PBMC) from healthy donors.^39^ Considered that salinomycin is a cation ionophore and can affect cellular Na^+^, K^+^, Ca^2+^ exchange, they introduced the K^+^ ionophore nigericin and Ca^2+^ ionophore thapsigargin and revealed that Ca^2+^ ionophore notably inhibited Fzd5 activated reporter, unlike the case for salinomycin and nigericin. The suppression of Wnt pathway as salinomycin's recognized effect has also been reported by several other research groups.^40^, ^79^ Additionally, the inhibitory effects of salinomycin on cancer‐related signaling pathways including MAPK pathway,^80^, ^81^ mTORC1 signaling,^40^ and Notch signaling^71^ have been also reported thereafter.

Lu group recently extended their work and demonstrated that salinomycin could also suppress Wnt signaling by targeting the β‐catenin/T‐cell factor 4E (TCF4E) complex.^82^ In line with this finding, our lab observed that salinomycin significantly reduced the protein kinase A (PKA) phosphorylated β‐catenin level in NB cells (unpublished data). Phosphorylation at Serine 675 of β‐catenin by PKA has been reported to be associated with the activation of downstream TCF/lymphoid enhancer factor transcription, and does not affect β‐catenin degradation.^83^, ^84^, ^85^ In a pulmonary fibrosis research, Hou et al. found that inflammation may trigger M2 macrophages to induce myofibroblast differentiation of lung resident mesenchymal stem cells (LRMSCs) via Wnt/β‐catenin signaling and salinomycin profoundly inhibited this differentiation, confirming the role of salinomycin as a Wnt/β‐catenin inhibitor.^86^

### 2.5 Sequestration of iron in lysosome

Intracellular iron (Fe^2+^) is tightly regulated to maintain iron homeostasis. Rodriguez group observed significant iron change in the established CD24^low^ breast cancer CSC population. They then synthesized a fluorescent amine derivative of salinomycin (ironomycin, also called AM5) and explored the effects of salinomycin and AM5 on iron homeostasis in CSC‐high cancer cell lines. As a result, salinomycin (0.5 μM) treatment led to the accumulation of iron in lysosome, decreased the expression of iron keeper ferritin and increased the level of iron regulatory protein 2 (IRP2), which normally inhibits the translation of iron transporter ferroportin. Thus, they proposed a novel inhibitory mechanism of salinomycin on breast CSCs that is through sequestering iron in lysosome.^44^, ^87^ The accumulation of iron in lysosome results in ROS production, which could induce cell death. Additionally, AM5 showed a ~10‐fold higher efficacy against HMLER CD24^low^ cells compared with salinomycin and meantime maintained the selectivity of CSC‐high cell population over CSC‐low cells. Moreover, AM5 displayed a significantly high cytotoxicity against the aldehyde dehydrogenase (ALDH) positive subpopulation of breast cancer cells, which is also well‐known as a cell subset with CSC trait.^88^, ^89^, ^90^ The antitumor effects of salinomycin and AM5 were further confirmed in two patient‐derived xenograft (PDX) models. Their work highlighted a more potent anti‐CSC efficacy of AM5 with low toxicity to normal cells and the promise of AM5 as a potential therapeutic drug. Since AM5 modified the original property of salinomycin as a monovalent ionophore and the intracellular distribution of AM5 (lysosomes) is different from that of salinomycin (cytoplasm), the actual mechanism of salinomycin's anti‐CSC action might be a bit more complicated than AM5.

### 2.6 Intracellular binding targets

Studies from us and also other groups have shown that salinomycin suppresses CSCs in many cancer types including breast cancer,^24^, ^34^, ^44^, ^75^, ^91^, ^92^, ^93^, ^94^ neuroblastoma,^51^ GBM,^38^, ^43^, ^76^, ^95^ medulloblastoma,^71^ pancreatic cancer,^20^, ^37^, ^96^ colon cancer,^67^, ^82^, ^97^, ^98^, ^99^ prostate cancer,^100^, ^101^, ^102^ melanoma,^103^ lung cancer,^72^, ^104^ and so on. Despite the above‐mentioned efforts, the cellular target of salinomycin remained unclear for a long time. Before the era of small‐molecule inhibitor design comes, conventional natural compound‐derived drugs discovered by chance lack of knowledge of their binding targets, such as thalidomide and salinomycin. Thalidomide's binding target has been identified through affinity purification using ferrite‐glycidyl methacrylate beads.^105^, ^106^ In addition, a cellular binding target of anticancer agent resveratrol has also been identified by drug affinity responsive target stability (DARTS) assay.^107^, ^108^, ^109^ By utilizing an integrated strategy combining DARTS method and coimmunoprecipitation (co‐IP) cross confirmation, our research group identified that NCL is likely a functional cellular binding target for salinomycin.^51^ We have demonstrated that salinomycin effectively inhibits NB growth with an IC_50_ significantly lower than that found with most currently used chemotherapeutic drugs for NB, for example, carboplatin.^110^ We also determined that salinomycin disrupts CD34 expression in NB via NCL, the elevated levels of which in NB tumors are associated with poor prognosis.

NCL is a multifunctional protein and is somewhat required for cell proliferation and growth. The binding of NCL to RNA, DNA and also many proteins has been widely reported. For instance, NCL regulates Matrix Metallopeptidase 9 (MMP9) mRNA translation after iron chelator treatment.^111^ NCL regulated CD133, CD34 expression in hematopoietic stem/progenitor cells (HSPC) by binding to their promoter regions.^112^, ^113^ Interestingly, almost at the same time we published NCL as a binding target of salinomycin, Reister et al. showed that NCL promotes Wnt signaling in HSPCs,^114^ which therefore attracts us to address whether the Wnt suppressing effect of salinomycin occurs through binding to NCL in our future study. Our research on the cellular target of salinomycin may also contribute to its future translation to clinical application, potentially stratify patients who are versus are not responsive to salinomycin therapy based on the expression of NCL. Another interesting study is that Park et al. attempted to detect the difference of ion channels between CD133^+^ and CD133^–^ NB cells. They found higher expressions of BKCa, Ca_v_1.3, and Na_v_1.7 proteins in CD133^+^ enriched NB‐CSCs, the addition of the Ca^2+^ ionophore ionomycin enhanced a stable Ca^2+^ influx.^115^ BKCa is also known to be expressed higher in excitable cells and could promote cancer cell growth and metastasis.^116^, ^117^, ^118^, ^119^ From another study, the expression of human ether‐a‐go‐go‐related gene (hEGR) K^+^ channel is observed in a subpopulation (CD34^+^CD38^–^CD123^high^) of leukemia cells but not in normal bone marrow CD34^+^/CD38^–^ HSPCs.^120^ Therefore, the question that if ion channel abnormality is a general feature of CSCs, and how NCL mediated the MoA of salinomycin against this feature of CSCs are warranted to be further investigated.

## 3 TOXICITY

### 3.1 In animals

In general, salinomycin's actions can be very dose‐dependent, species‐dependent, and cell type‐dependent.^26^, ^121^ Salinomycin is already an approved drug by the United States Food and Drug Administration (FDA) for use in animal feeds.^122^ At a normal feeding dosage, salinomycin does not cause noticeable abnormalities in animals. Whereas, accidental overdose and mixed overfeed of salinomycin have been reported to cause death in adult turkeys,^123^ horses,^124^ dairy calves,^125^, ^126^ pigs,^127^ sheep,^128^ rabbits,^129^ cats,^130^ and so on. For example, 60 mg/kg body weight (BW) salinomycin feed for chickens is safe and does not raise significant adverse effects while 120 mg/kg BW salinomycin may affect the immune system of the chicks.^131^ In the case of cats, van der Linder‐Sipman et al. performed clinical and pathologic examination, which indicated a distal polyneuropathy in poisoned cats.^130^

Miyasaki et al. reported that an acute toxicity of salinomycin was examined in mice with LD_50_ (lethal dose, 50%) of 18 mg/kg intraperitoneally and 50 mg/kg orally.^31^ In the work by Boehmerle et al., it addressed the intoxication of salinomycin in mice and reported that at the dose of 5 mg/kg, salinomycin is well tolerated. Pharmacokinetics evaluation showed that salinomycin was almost completely eliminated within 5 h after injection. No side effect was observed from detecting BW and blood markers, while peripheral polyneuropathy was observed in salinomycin‐treated mice. In another study, Resham et al. studied the in vitro drug metabolism and pharmacokinetic parameters of salinomycin and observed that salinomycin undergoes rapid metabolism in liver microsomes and has a high intrinsic clearance, which is mainly mediated by cytochrome P450 (CYP) enzymes, especially CYP3A4. Notably, the percentage of salinomycin in ultracentrifuged unbound fraction of plasma in human was higher compared with mouse and rat plasma, indicating that its metabolism may be faster in human.^132^

The combination of Na^+^/Ca^2+^ exchanger inhibitors together with salinomycin prevented the neuropathy without affecting salinomycin's anticancer efficacy.^133^ In Gupta's work, they observed a significant anticancer effect of salinomycin in breast cancer cell inoculated mice at the dose of 5 mg/kg BW.^24^ Ojo et al. investigated the toxic effect of salinomycin, if any, on fertile ability in male mice. They tested the doses of 1, 3, or 5 mg/kg administered daily for 28 days. Decreased motility and spermatozoa count were once observed; however, spermatogenesis was observed again in testis 28 days after salinomycin withdrawal, indicating a reversible dose‐dependent adverse effects of salinomycin on male reproductive system of mice.^134^, ^135^

### 3.2 In human cells

Literature revealed that the EC_50_ values of salinomycin against tumor cells vary across cancer cell types. For instance, from our previous work, the effective concentration of salinomycin eliminating CSC‐high NB cells is about 1–2 μM in less than 48 h in vitro^51^; the EC_50_ of salinomycin killing CSC‐high GBM cells is around 1.25 μM^76^; the IC_50_ of salinomycin against medulloblastoma cells is raging from 0.1 to 2 μM^71^; and the EC_50_ of salinomycin on CSC‐high pancreatic cancer cells is approximately 0.5–2 μM (from our unpublished data). Boehmerle et al. tested the potential neurotoxic effect of salinomycin on human dorsal root ganglia and Schwann cells (neural cells) and reported that at a high dose (10 μM) it could induce Na^+^ influx and a subsequent Ca^2+^ influx, which may consequently induce calpain and cytochrome c‐mediated cell death. Na^+^/Ca^2+^ exchanger (NCX) inhibition significantly antagonized high‐dose salinomycin‐induced peripheral neuropathy on these cells.^136^ In a study that tested salinomycin's effect on Leukemia cells, Fuchs et al. found that salinomycin‐induced apoptosis of human CD4^+^ T‐cell leukemia cells but not normal CD4^+^ T cell at effective dosage.^137^

Considered from one of the CSC origin theories that hypothesized CSCs may be developed from human bone marrow mesenchymal stem cells (hBMSC), Scherzed et al. investigated the functional impairment of hBMSC by salinomycin in vitro and reported that cytotoxic effects of salinomycin were observed at concentrations of 30 μM and above after 24 h treatment. They observed no adverse effects on the essential functional properties, the immunophenotype and multidifferentiation capacity of hBMSC.^138^ They also investigated and summarized the geno‐ and cyto‐ toxicity of salinomycin for human nonmalignant cells.^139^ After testing the cytotoxic effects of salinomycin on primary human nasal mucosa cells and peripheral blood lymphocytes collected from 10 individuals. They reported that no genotoxic effects were observed, while cytotoxic effects in nasal mucosa cells and lymphocytes at concentrations of 10–20 μM and above were observed. A slight elevation of interleukin 8 (IL‐8) secretion was observed at 5 μM, which may indicate a proinflammation activating potential of salinomycin.^140^, ^141^ Notably, Szkudlarek‐Mikho et al. reported an inhibitory effect of salinomycin on adipogenesis. The suppression of preadipocytes differentiation into adipocytes was observed at a concentration of 10 nM and above, which appears not associated with apoptosis induction or cell proliferation disruption. The finding revealed the potential role of salinomycin as antiobesity agent and a harbinger of its toxicity on the adipose tissue.^142^ Furthermore, Scherzed et al. later tested the effect of 4‐week chronic exposure of hBMSCs to low dose (0.1 μM) salinomycin in vitro and observed a moderate suppressing effect on the migration ability of hBMSCs with no alteration seen on the cytoskeletal structure.^143^

In light of above studies, salinomycin‐induced cytotoxic and proinflammatory effects were seen at concentrations ~fivefold higher and ~twofold higher than that relevant to anticancer treatment, whereas the suppression of cell differentiation was observed at a low dose. These studies have not involved other human cells such as liver, kidney, and muscle cells, adverse effects in nonmalignant cells need to be monitored. The dose‐dependent manner of salinomycin elicits that the definition of a therapeutic index and the selection of administration method are of pivotal importance, considering its potential safety risk.

## 4 CLINICAL STUDIES

Preclinical studies are anticipated to contribute to an ultimate clinical utility. To date, the only clinical cases of salinomycin application are documented by Naujokat et al. in Germany.^144^ The authors used salinomycin for a 40‐year‐old female patient with triple negative metastatic breast cancer who showed no response to conventional treatment. Their result showed that tumor metastasis was markedly regressed after 12 cycles intravenous administration of 200 μg/kg salinomycin every second day. Similar results of salinomycin's regression on tumor growth and metastasis were observed in other three patients with metastatic breast cancer, one patient with metastatic ovarian cancer, and one patient with metastatic head and neck squamous cell carcinoma. They also used a combination of salinomycin (200 μg/kg) with erlotinib for an 82‐year‐old female patient with advanced and metastatic squamous cell carcinoma who showed no response to conventional treatment. After 14 cycles of combination therapy, a significant tumor regression was observed. Since tumor progressed again after 3 months and erlotinib showed adverse effect on the patient, a further 12 cycles of intravenous administration of 250 μg/kg salinomycin every second day. The patient was monitored for 4 months after treatment and showed stable disease status with no progression.

## 5 CHEMICALLY MODIFIED SALINOMYCIN DERIVATIVES AND SYNTHESIZED CONJUGATES

Along with the accumulated mechanistic understandings of salinomycin's activities, chemical modification of salinomycin emerges as an interesting research direction in pursuit of both increasing its anticancer activity and decreasing its potential toxicity, that is, developing agents with a higher therapeutic index.^145^ The chemical structure of salinomycin was identified by Miyasaki et al. as a ployether carboxylic ionophoric antibiotics^31^ with a molecular weight of ~751 Da.^31^, ^132^, ^144^ There is one carboxylate residue at C1, and several hydroxy (OH) residues in salinomycin important for ionophoric transport. Acylated OH slightly decreased K^+^ transportation rate of salinomycin but esterified COOH (C1) made it lose its ionophoric activity.^53^ Work on the modification of salinomycin to enhance its anticancer activity has generated more than 100 derivatives, via either structure modification or dimer synthesis.^44^, ^47^, ^48^, ^49^, ^77^, ^146^, ^147^, ^148^, ^149^, ^150^, ^151^, ^152^, ^153^, ^154^, ^155^, ^156^, ^157^, ^158^, ^159^, ^160^, ^161^, ^162^, ^163^, ^164^, ^165^, ^166^, ^167^, ^168^, ^169^, ^170^ Of note, derivatives with single or double modification of C1 and/or C20, or double modification of C17/C21, or dimers connected at C20 showed a noteworthy improvement in terms of anticancer activities.^145^

Huczyński group made great efforts on the modification of salinomycin's structure to obtain salinomycin derivatives (SARs) and the synthesis of salinomycin conjugates with other drug or compound (SAXs) to enhance its activities, including antimicrobial, antiprotozoal, antiparasitic and antiproliferative activities.^26^, ^77^, ^146^, ^147^, ^148^, ^149^, ^150^, ^151^, ^152^, ^153^, ^155^, ^156^, ^157^, ^158^, ^170^, ^171^, ^172^, ^173^, ^174^, ^175^, ^176^, ^177^, ^178^, ^179^, ^180^, ^181^, ^182^, ^183^, ^184^, ^185^ For instance, by semi‐synthesis they generated series of amide derivatives and ester derivatives at C1 position; by “click” chemistry they synthesized salinomycin conjugates with the anticancer drug floxuridine (FdU or 5‐FU) (SAX1) or with the anti‐HIV drug 3'‐azido‐3'‐deoxythymidine (AZT) (SAX2). Recent reviews published by Antoszczak comprehensively summarized the recent work on salinomycin derivatives.^145^, ^170^ Notably, the improvement of the efficacy of these derivatives or conjugates against different cancer types varies. For example, a C1 ester derivative (SAR1) sensitizes doxorubicin‐resistant LoVo colon cancer cells by almost eightfolds while shows a similar effect to that of salinomycin against vincristine‐resistant HL‐60 leukemia cells,^145^ indicating its potential of cell‐dependent use in future. A following study on this C1 ester derivative revealed that it has a twofold improved anticancer activity against a leukemia cell line compared with that of salinomycin, although unlike salinomycin it did not achieve a synergistic effect when used together with Bcl‐2 inhibitor.^155^ The salinomycin‐FdU (SAX1) conjugate inhibits the proliferation of human cancer cell lines at a similar concentration to salinomycin, however its toxicity to normal murine fibroblast cells BALB/3T3 reduced more than twofolds, giving an improved selectivity index (SI).^156^

Antoszczak and colleagues also attempted to synthesize ionophore dimer hybrids. Of all the dimers they generated, the salinomycin‐monensin (MON) dimer (SAX3) and a salinomycin dimer (SAL‐dimer2) give similar IC_50_ values to that of salinomycin but increase ~three‐ to fourfolds for their SIs after tested in MCF‐7 breast cancer cells and MCF‐10A normal breast cells.^177^ They recently further derived a series of tertiary amides of salinomycin and their C20‐oxo analogues, a C1 tertiary amide (SAR2) salinomycin showed an improved SI index when tested against a triple negative breast cancer cell line (MDA‐MB‐231); the C20 ketone derivative (SAR7) displayed cytotoxic effects in an ex vivo model of breast cancer comparable to salinomycin, although did not stand out from in vitro cell‐based assays.^173^ In parallel, Rodriguez group also generated this derivative and observed a lower IC_50_ against EMT in CSC‐high breast cancer cells in vitro and a higher selectivity over CSC‐low counterpart cells in comparison to salinomycin, though the toxicity to normal cells was not tested.^186^

At the same time, Wu group synthesized salinomycin diastereoisomers at C17 and C21 and their benzoylated derivatives, after tested their antiproliferative effects on colon cancer and breast cancer cell lines as well as rat cerebral cortex neuron cells in vitro, the 17,21‐di‐epi‐20‐O‐Bz‐salinomycin sodium salt (SAR15) showed a nearly twofold improvement and a notably better therapeutic intex.^167^ Later after that work, they synthesized salinomycin‐hyaluronic acid (HA) conjugates and tested their antiproliferative activities against several cancer cell lines. The results showed that conjugates led to an activity improvement in comparison with the effect of physical mixture of HA and salinomycin, especially three C1 hydroxamic acid conjugates (SAR3‐5) showed two‐ to threefold higher efficacies in a colon cancer cell line (HT‐29), a metastatic gastric cell line (HGC‐27) and a triple negative breast cancer cell line (MDA‐MB‐231) compared with salinomycin.^145^, ^159^, ^166^ However, the toxicity to normal cells of these conjugates were not tested in that study.^159^ In addition, as salinomycin displays a poor water soluble capability (17 mg/L), derivatives with improved aqueous solubility have also been reported by Awad aiming to promote the efficiency of drug delivery to cancer cells.^187^ The cancel cell cytotoxicity was observed at two‐ to threefolds higher than that of salinomycin, while no data were showed regarding the effect of the derivative against normal cells. The tests of these derivatives and conjugates were mainly performed in mixed cancer cell populations such as drug‐resistant cell lines and ex vivo tumor tissues, the improvement of these derivatives on CSC selectivity is yet unknown.

Salinomycin is also be reported by the Strand group to be associated with the transport of polar alkali metals via lipophilic membranes,^47^, ^48^ the free carboxylate at C1 is also functionally required, in agreement with Miyazaki et al.'s work. Of importance, Strand group showed that acylation of the C20 hydroxyl resulted in a reduction of its IC_50_ values by 80% against breast cancer cells.^161^ This result elicits the importance of C20 for salinomycin exhibiting cytostatic activity. They later summarized the structure–activity relationship of salinomycin and pointed out that C11 and C1 are essential ion coordinating motifs and C20‐O‐acylated analogs (SAR8‐11) give potentiated anticancer activities both antiproliferatively and CSC‐selectively.^48^, ^49^ Moreover, the treatment of salinomycin or its C20‐O‐analogs inhibited the migration of breast cancer cells, decreased the expression of vimentin, which is the marker of mesenchymal phenotype, and increased the level of E‐cadherin, which is the marker of epithelial phenotype, suggesting that salinomycin and its derivatives induced a mesenchymal‐to‐epithelial change and therefore may prevent metastasis.^49^

Thereafter, Kamlund et al. did an interesting experiment to track individual breast cancer cell division instead of changes in cell population with and without 0.5 μM salinomycin treatment using digital holographic microscopy. Their results showed that after 24–48 h treatment of salinomycin, cell proliferation was dramatically inhibited, the number of cells staying undivided increased and cell motility/migration evidently decreased. While, in a 72–96 h span, approximately 3%–5% tracked cells remain slowly proliferating.^188^ Recently, on the basis of a semi‐synthesized C20‐O‐analog (SAR11) with a markedly enhanced anticancer activity (~10‐fold), Strand group further generated a fluorescent conjugate of the analog using nitrobenzoxadiazole (NBD) reporter (SAR12 or SAL‐NBD). Although the working concentration of this conjugate is similar to that of salinomycin, by using fluorescence detection technology, they revealed its biological ability to act in the ER membrane of breast cancer cells to cause stronger Ca^2+^ release from ER into the cytosol, which may be mediated via a counter‐flux of K^+^ ions.^50^ More recently, with their gained knowledge that the acylation at C20 position and the esterification/amidation at C1 moiety benefit the potentiation of salinomycin's activity, they generated a series of doubly modified derivatives. However, after testing their efficacy in cancer cell lines and ex vivo breast tumor tissues, few candidates stood out with markedly improved cytotoxicity compared with the original salinomycin.^77^

**Figure 3:**
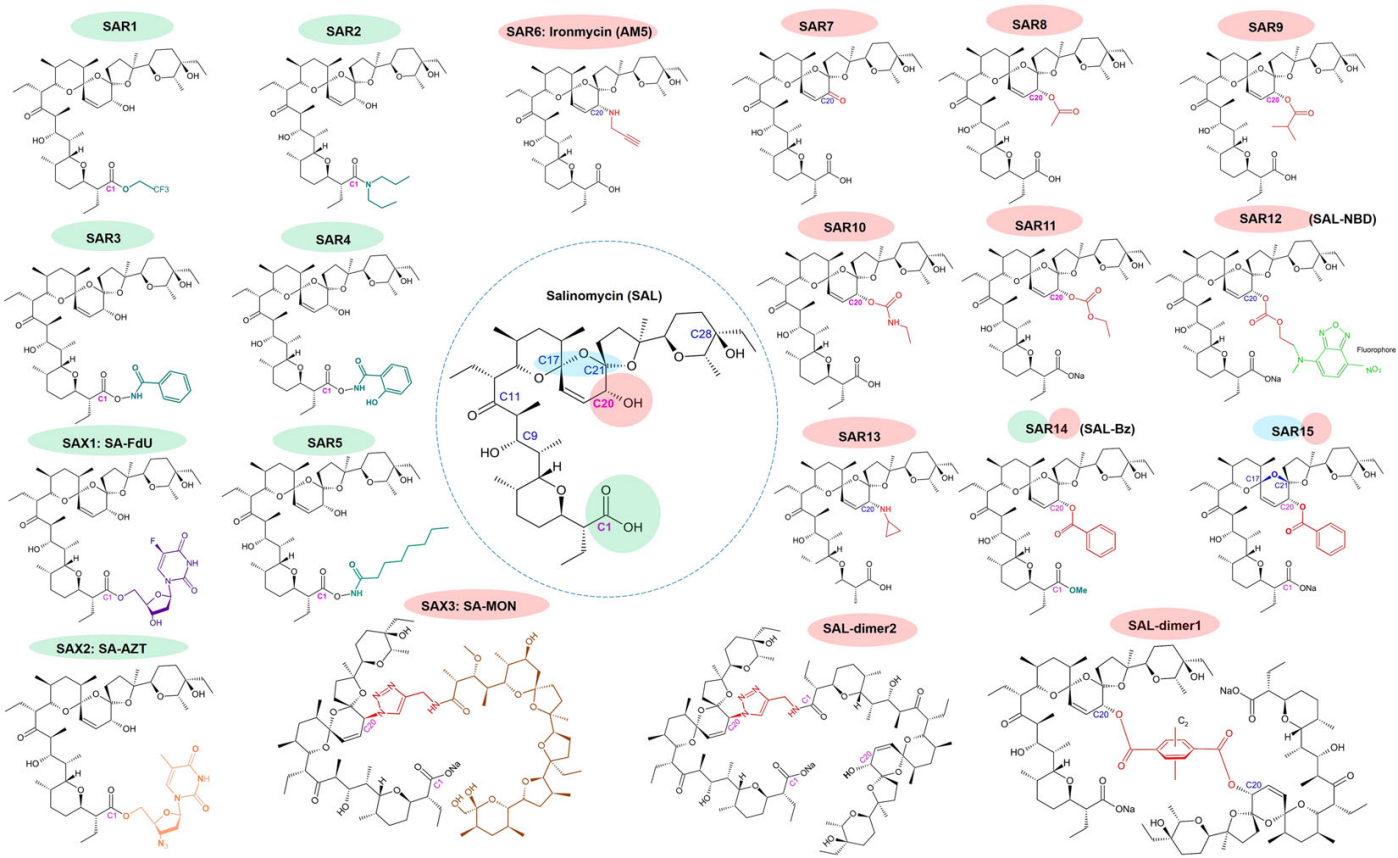
Promising reported derivatives and conjugates of salinomycin. Chemical structures were drawn using ACD/ChemSketch software. Literature of all structures was cited in the text. Red circles: modifications on the C20 position of salinomycin; green circles: modification on the C1 position of salinomycin; blue circles: modification on the C17 and C21 positions of salinomycin [Color figure can be viewed at wileyonlinelibrary.com]

The above‐mentioned amine derivative AM5 (ironmycin) synthesized by Mai et al. from Rodriguez group, showing a higher cytostatic activity on breast cancer CSCs and a lower toxicity on normal breast cells compared with salinomycin, is also generated from modification at C20.^44^, ^87^ This study group recently further synthesized a series of single or multiple modified salinomycin derivatives. A C20 cycloalkyl‐modified derivative (SAR13), with a functional similarity (iron sequester) and a slight structural difference to AM5, also shows a promising potency and selectivity against CSC‐high breast cancer cells, which warrants the future investigation on toxicity against normal cells and in vivo studies.^186^ Moreover, Lim et al., in their work of testing the synergistic effect of salinomycin on GBM, synthesized shortened salinomycin derivatives (split at C9), and C1‐Me/C20‐Bz double modified analog. The shortened salinomycin parts totally lost the anticancer activity, while the C1‐Me/C20‐Bz derivative (SAR14 or SAL‐Bz) was demonstrated to have a higher potency in an orthotopic GBM mouse model than the original salinomycin and displayed favorable synergistic effects with radiation resulting in a much longer survival of tested mice. Similar to other groups' work, C1/C20‐modified derivatives of salinomycin have the upmost attractive potency for future studies toward ideal anticancer agents.^38^ The promising derivatives with higher cytostatic efficacies than salinomycin were enumerated in Figure 3.

## 6 SALINOMYCIN‐BASED DRUG DELIVERY STUDIES IN THE ERA OF NANOMEDICINE

In addition to the works on the modification of salinomycin structure chemically, there are also research trying to potentiate salinomycin's efficacy by physically improving drug delivery with nano‐drug delivery systems (DDS).^33^, ^145^ The first application of drug‐delivery system in clinical trials is in the 1990s,^189^ which is a doxorubicin‐liposome agent for the patients with AIDS‐related Kaposi's sarcoma. Nanocarriers are designed to overcome the disadvantages of chemodrugs, such as side effects, low solubility, short cycle time and insufficient target effect, and improve their therapeutic index.^190^, ^191^, ^192^, ^193^, ^194^, ^195^ Current reported nano‐materials include liposomes, polymer‐based micelles, and metal nanoparticles,^192^, ^196^, ^197^ which have been used in salinomycin delivery system studies taking their advantages of low toxicity and good biocompatibility in vivo.^198^, ^199^, ^200^, ^201^, ^202^, ^203^, ^204^, ^205^, ^206^, ^207^, ^208^, ^209^, ^210^, ^211^ The concept for the design of a salinomycin‐based cancer cell targeted DDS can be at least twofold: (1) salinomycin has a higher selective efficacy on stem‐like cancer cell populations compared with other cancer cells, for example, CD133^+^ pancreatic cancer cells or GBM cells, CD44^high^/CD24^–/low^ or ALDH^+^ breast cancer cells, CD34^+^ neuroblastoma cells. Therefore, the construction of DDSs that can specially target and deliver salinomycin to these populations by integrating antibodies or aptamers against certain surface markers is expected to efficiently eradicate the hinder quiescent CSCs; (2) salinomycin is capable to sensitize cancer cells to conventional clinical treatment methods, both radiation and chemodrugs (e.g., doxorubicin, gemcitabine, and gefitinib). Therefore, the construction of DDSs that can deliver salinomycin together with certain chemodrugs or monoclonal antibodies simultaneously may have the hope of removing both therapy‐resistant CSCs and proliferative non‐CSC tumor bulk, which may thereby prevent recurrence.

Wei et al. generated a salinomycin delivery method by conjugating it to a HA‐based nanogel to target CD44^+^ multidrug resistant cells and yielded two to seven times higher efficacy against those cells.^201^ Choi et al. developed a multitarget delivery system by using the combination of reduced graphene oxide–silver nanoparticle nanocomposites (rGO–Ag) and salinomycin.^202^ This combination delivery system induced cytotoxicity and caspase‐dependent apoptosis in ovarian CSCs and showed fivefold higher efficacy on ALDH^+^/CD133^+^ cells in comparison to salinomycin alone.^202^ Yi et al. constructed a salinomycin‐loaded PLGA nano‐carrier attaching CD133 antibodies to target ovarian CSCs, which displayed improved therapeutic effects compared with salinomycin alone.^212^ In addition to antibodies, DNA aptamers have been widely used in DDS constructions, as they are small, stable, and easily synthesizable.^213^, ^214^, ^215^ For example, Zeng et al. developed salinomycin‐based nanoparticles (NP) with anti‐CD20 aptamer targeting CD20^+^ melanoma CSC subsets. Administration of these NPs in melanoma xenograft mice showed a superior efficacy in inhibiting tumor growth.^199^ Jiang et al. generated PLGA‐NPs conjugated epidermal growth factor receptor (EGFR) and CD133 aptamers with salinomycin to target hepatocellular carcinoma (HCC) cells that simultaneously expressing EGFR and CD133. As expected, these NPs showed higher cytotoxicity in CD133^+^ HCC cells and improved tumor inhibition in mice xenografts compared with salinomycin alone.^198^ Similar to this study, Zhou et al. also constructed salinomycin sodium NPs cotargeting EGFR and CD133 expressed in lung cancer cells and observed an improved tumor growth inhibitory effect in mice xenografts.^216^

Zhao et al. applied elastin‐like polypeptide to construct NPs for a salinomycin derivative (4‐(aminomethyl) benzaldehyde‐modified salinomycin aiming to develop an immune tolerant approach to monitor metastasis in orthotopic murine models.^203^ The NPs suppressed metastasis in 4T1 orthotopic breast cancer mouse model. In addition, the combined use of salinomycin NPs and paclitaxel NPs effectively retarded primary tumor growth and improved overall survival. Tsakiris et al. recently generated SN38 (a chemodrug) and salinomycin lipid nanocapsules and the coadministration of them evidently prolonged the survival times of mice bearing colon cancer compared with single drug.^217^ Wang et al. showed that, compared with free salinomycin, the salinomycin nanocrystals they generated had a two times higher anti‐colon tumor effect in APC^min/+^ transgenic mice.^218^ Notably, a pharmaceutical company recently announced that they were granted by FDA to study salinomycin nanoparticle (HSB‐1216) in small cell lung cancer, which is currently under ex‐US (Germany) phase I clinical trial phase.^219^, ^220^ HSB‐1216 is generated by encapsulating salinomycin within QUATRAMER delivery technology, which used a polyethylene glycol (PEG)‐polypropylene glycol (PPG)‐PEG modified polylactic acid (PLA)‐tetra‐block copolymer. The team reported that HSB‐1216 has strong effects on eliminating small cell lung CSCs in mice^221^ and also displays potent inhibitory effects on tumor biopsy‐derived organoids from triple negative breast cancer patients.^222^

## 7 PERSPECTIVES

Salinomycin has been used as a poultry medicine for a long time and found to be an anticancer agent since 2009. Mounting data have been published in various cancer types regarding its anti‐CSC activity from preclinical research both in vitro and in vivo.^223^ However, it has not been widely used clinically in humans. Of note, there are several pieces of promising clinical cases in advanced cancer regression using salinomycin as described above,^144^ but its use has not yet been continued in the treatment of other cancers. In terms of safety based on current data, salinomycin does not cause severe toxicity to normal cells at the dose against CSCs, whereas high dosage or longtime expose at low dose may cause neural toxicity and differentiation inhibition on normal mesenchymal stem cells.^27^, ^42^, ^138^, ^139^ Slight inhibitory effects on fertile ability were observed in male mice during salinomycin treatment; however, spermatogenesis was observed again 28 days after salinomycin withdrawal.^134^, ^135^ The derivative and conjugate synthesis of salinomycin alone or integrating other compounds/chemodrugs and the development of nano‐drug delivery systems encapsulating salinomycin or drug cocktails hold the hope to improve the therapeutic index of salinomycin significantly and ultimately develop a series of clinically useful anticancer agents.

As one component of TME, neurological regulation was also closely associated with cancer, especially visceral cancers, such as pancreatic ductal adenocarcinoma.^224^, ^225^, ^226^ The effect of salinomycin on TME or host immune defense system has not been much studied. One recent work by Ebokaiwe et al. explored the role of salinomycin on indoleamine 2,3 dioxygenase (IDO), which is active in many tumor types and may promote tumor immune tolerance.^227^ Their results showed that salinomycin treatment lowered the levels of IDO1 and IDO2, inhibited interferon gamma (IFN‐γ)‐mediated Janus kinase /signal transducer and activator of transcription (JAK/STAT) and nuclear factor kappa B (NF‐kB) pathways, and restored the proliferation of T cells in vitro.^227^ Another study by Shen et al. showed that low‐dose treatment of salinomycin‐mediated repolarization of tumor‐ associated macrophage (TAM) and neutralized interleukin 4 (IL4)‐induced inflammation in murine breast cancer cells, and inhibited tumor growth and pulmonary metastasis in tumor‐bearing mice.^228^ These pieces of work open another gate of research direction of salinomycin in light of the regulatory roles of TME in tumor development and progression.

Considering that the antitumor effect of salinomycin in human cancerous tissues could be different from in vitro cell‐based assays, more work using orthotopic models, humanized models, patient tumor originated cells or ex vivo models are needed to better evaluate the anticancer efficacy and intoxication of salinomycin, its derivatives/conjugates and its embedded nanoparticles. Additionally, in‐depth knowledge of salinomycin's MoAs is still needed to fully understand not only its cytostatic activities on tumor cells or separate normal cells but also a comprehensive response of both tumor and its surrounding microenvironment. Furthermore, we think that insufficient assessment of CSC status might be an important player in treatment failure and recurrence. Thus, research on deciphering the explicit mechanism of CSCs is of importance as it would contribute to the better definition of CSC populations, prompt novel discovery of CSC targeting therapeutics, and help translate salinomycin to the clinic, either itself or as a partner in combination therapy.

## CONFLICT OF INTERESTS

The authors declare that there are no conflict of interests.

## Data Availability

Data and materials are available from the corresponding author Dr. Erxi Wu upon request. E‐mail: Erxi.Wu@BSWHealth.org.
